# Traditional Chinese Medicine-Based Network Pharmacology Could Lead to New Multicompound Drug Discovery

**DOI:** 10.1155/2012/149762

**Published:** 2012-12-17

**Authors:** Jian Li, Cheng Lu, Miao Jiang, Xuyan Niu, Hongtao Guo, Li Li, Zhaoxiang Bian, Na Lin, Aiping Lu

**Affiliations:** ^1^School of Basic Medical Sciences, Beijing University of Chinese Medicine, Beijing 100029, China; ^2^Institute of Basic Research of Clinical Medicine, China Academy of Chinese Medical Sciences, Beijing 100700, China; ^3^School of Chinese Medicine, Hong Kong Baptist University, Kowloon, Hong Kong; ^4^Institute of Materia Medica, China Academy of Chinese Medical Sciences, Beijing 100700, China

## Abstract

Current strategies for drug discovery have reached a bottleneck where the paradigm is generally “one gene, one drug, one disease.” However, using holistic and systemic views, network pharmacology may be the next paradigm in drug discovery. Based on network pharmacology, a combinational drug with two or more compounds could offer beneficial synergistic effects for complex diseases. Interestingly, traditional chinese medicine (TCM) has been practicing holistic views for over 3,000 years, and its distinguished feature is using herbal formulas to treat diseases based on the unique pattern classification. Though TCM herbal formulas are acknowledged as a great source for drug discovery, no drug discovery strategies compatible with the multidimensional complexities of TCM herbal formulas have been developed. In this paper, we highlighted some novel paradigms in TCM-based network pharmacology and new drug discovery. A multiple compound drug can be discovered by merging herbal formula-based pharmacological networks with TCM pattern-based disease molecular networks. Herbal formulas would be a source for multiple compound drug candidates, and the TCM pattern in the disease would be an indication for a new drug.

## 1. Introduction

Completed in 2003, the human genome project plunged the world into the postgenomic era aimed at understanding the global function of the genome through systems biology, mathematics, and computational techniques [1]. The large-scale generation and integration of genomic, proteomic, signaling, and metabolomic data suggest that most diseases are much more complex than initially anticipated and that most disease genes vary in their expression patterns [2]. Besides, organic life is a nonlinear system involving all kinds of interactions between networks of biomacromolecules, cells, drugs, and each other [3], and the “one drug for one gene for one disease” model failed to work because one drug often has many targets, and many protein targets are targeted by more than one drug [4, 5]. These insights triggered a major change in the strategies adopted in the new drug discovery: the shift from single compound drugs to multiple compound drugs. Based on these concerns, network pharmacology emerged as the next paradigm in new drug discovery with its elucidation capacity in the complexity of biological process [6]. Network pharmacology, a system biology-based methodology, is a new approach to drug design that encompasses the construction of disease networks, drug-target networks, and drug-disease networks. In the network pharmacology-based new drug discovery, a biological network of a disease and a pharmacological network of the candidate are crucial since the candidate showing the well-matched its pharmacological network with some certain disease biological network, would be developed into a new drug [7, 8].

Multiple component drugs, considered as a future direction for new drug discovery, have received widely spread reported [9–11]. Polypill was reported to be an alternative for real therapeutics [12, 13]. Traditional chinese medicine (TCM), with its unique theory and long history, identifies the patients with Bian Zheng (also called pattern or syndrome differentiation) which diagnoses the patients based on TCM information, such as symptoms, tongue appearances, and pulse feelings, and TCM treats the patients accordingly with herbal formulas (which contains multiple components) targeting to the TCM pattern [14]. In another word, TCM uses multiple compound herbal products to treat the pattern in the disease. Thus it is reasonable to suggest finding new multiple compound drugs from herbal formulas for treating a subgroup (TCM pattern) of the patients in a certain of disease.

The major hurdles in the multiple compound new drug discoveries are how to identify the TCM pattern in a disease and build up the pharmacological network of herbal formula. TCM pattern in a disease can be identified with biological network biomarkers, and the pharmacological network of herbal formula can be built up with newly network pharmacological approaches. Thus, by integrating the TCM pattern molecular network and the pharmacological network of herbal formulas, which we would like to call as TCM-based network pharmacology, could be a novel way to lead to multiple compound drug discoveries.

## 2. TCM Pattern-Based Disease Molecular Networks

Currently, the integration of TCM pattern classifications and biomedical diagnoses is becoming a common clinical diagnostic model in China and has produced better clinical outcomes [15]. As a case, clinical research on rheumatoid arthritis (RA) suggests that RA patients should be treated by different therapies based on their TCM patterns [16]. Thus, TCM pattern classification in a disease could be a more precise indication when designing and evaluating a drug candidate. As a diagnostic result in TCM pattern classification, TCM patterns could link up the corresponding subnetworks of a specific disease in the context of molecular medicine. The present information about a disease could be collected to establish molecular networks underlying the disease. For example, the molecular network of RA has been established [17, 18]. The network of a disease could help identify the corresponding pharmacological network for therapeutic intervention, by merging the disease molecular network and the intervention pharmacological networks. In recent years, many researchers have paid more attention to the molecular networks built on the TCM pattern in some diseases [19]. A wide variety of TCM pattern-based disease molecular network applications have already been reported and bridged the gap between TCM patterns of Chinese medicine and diagnostic parameters of western medicine for example, we and others have surveyed plentiful typical cold and hot TCM pattern patients and examined *omics* information, such as genomics [20–23] or metabolomics [24, 25]. According to the cold and hot patterns-based bionetwork we could not only open out the mechanism of TCM pattern, but also understand the complexity of life processes [26, 27]. Furthermore, at another example, the biochemical changes are identified in kidney deficiency syndromes animal model through chemometric analysis [28]. In such a case, the integration of next generation *omics* technique will yield fundamental insights into the TCM pattern-based disease molecular networks. Then, with the help of TCM pattern-based networks in a disease, it seems to not only translate between different diagnostic readouts in TCM and western medicine, but also discover potential drug candidates.

## 3. Herbal Formula-Based Pharmacological Networks

More studies have shown that herbal formulas are effective in treating some diseases. As the cases, Lam et al. reported that a four-herb Chinese medicine PHY906 could reduce gastrointestinal toxicity induced by chemotherapy drug CPT-11 through multiple mechanisms including inhibiting CPT-11-triggered inflammation, promoting intestinal recovery, and intestinal progenitor cell repopulation [29]; Wang et al. explored the molecular mechanism and synergistic effects of each ingredient in Realgar-Indigo naturalis formula (RIF), a well-known and clinically proven TCM formulae for leukaemia therapy, and found that arsenic in Realgar directly attacked the receptor on coprotein in leukaemia cells, Indirubin in Indigo antagonized the toxicity of arsenic and slowed leukaemia cell growth and Tanshinone in red sage root restored pathways that stop leukaemia from spreading [30]. However, the conventional methods are hard to elucidate the pharmacological mechanism for multiple compound containing herbal formulas, and it has been believed that systems biology could be helpful in pharmacological study. Using a metabolomic method of reversed-phase liquid chromatography/quadrupole time-of-flight mass spectrometry (LC-Q-TOF-MS), Jiang et al. reported that Shexiangbaoxin Pill (SBP) could be used to treat myocardial infarction (MI) through regulating the perturbed pathway of energy metabolism, and five biomarkers, including creatine, uridine, glutamate, oxalosuccinic acid and nicotinamide mononucleotide, were completely reversed to normal levels in MI rats administrated with SBP for 15 days [31]. Li et al. established a method called distance-based mutual information model (DMIM), and through which they demonstrated that six herbs in Liu-wei-di-huang (LWDH) formula connected closely with common responsive genes enriched in cancer pathways and neuroendocrine-immune pathways, and also LWDH formula-treated diseases shared an overlapped molecular basis associated with the angiogenic processes as well as the imbalance of the human body [32]. In this respect, systems biology could be helpful in not only pharmacological mechanism study, but also new drug discovery.

Though still fairly new, researchers have been trying to explore some new paradigm in drug discovery with the aid of pharmacology biology, a part of systems biology, in which comparative reverse systems pharmacology and drug-combination studies, guided by system response profiles (SRPs), might be the two effective ways [33]. Another strategy, targeting at the human genome-microbiome axis, might also become a novel ways to discover new drugs from traditional chinese medicine (TCM) using systems biology [34]. These newly developed pharmacological networks are not only used to explore the pharmacological activity of a single compound drug, but they can also be used to examine combination therapy (drug combinations) [35, 36]. TCM herbal formulas with multiple compounds are pharmacologically targeting biological networks, instead of single target. Aided by information from genomics, proteomics, and metabolomics, researchers are seeking a methodology to build molecular pharmacological networks for herbal formulas or combination therapy [37]. For example, Salvia miltiorrhiza (SM) and Panax notoginseng (PN) in combination (SMPN) have been widely used (primarily in TCM) for the treatment of coronary heart disease, and we combined text mining with bioinformatics to build functional networks for SMPN [38]. These results suggest that the pharmacological activity of SMPN is the outcome of the interactions between SM and PN in the multiple pathways and biological processes during the treatment of coronary heart disease. With the help of pharmacological networks, we would know more about the pharmacological activities of the multiple compound drug candidates from herbal formulas.

## 4. TCM-Based Network Pharmacology in Multiple Compound New Drug Discovery

**Figure 1:**
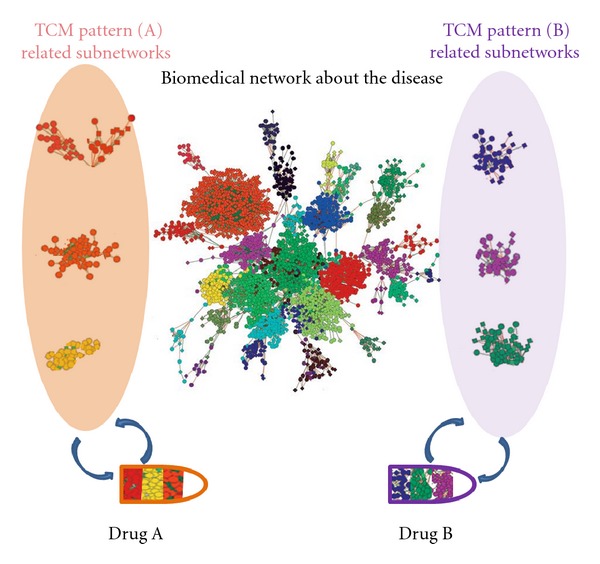
A diagram of drug discovery based on TCM-based network pharmacology. Each circle or diamond represents one gene or protein. Functionally interconnected genes or proteins are grouped by bioinformatics and shown in different colors. The biological networks in a disease include the general biological network of the disease (middle part) and TCM pattern network of the disease (Pattern (A) and (B) as an example). The shared disease biological networks and TCM pattern networks are shown in different colors. Drug A and drug B (in lower part) is targeting to the general biological network of the disease and molecular network of TCM pattern (A) and (B) in the disease respectively, and thus the drug A could be good for the disease with TCM pattern (A) and drug B could be good for the disease with pattern (B).

**Figure 2:**
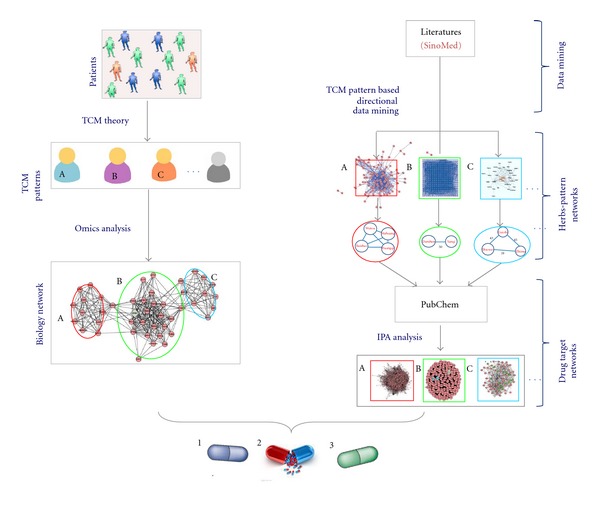
A conceptual model for multipl compound drug discovery using TCM-based network pharmacology. In the left of the paradigm, the molecular network of the disease-TCM pattern (lower left) can be constructed by analyzing the omics data from patients classified with the TCM pattern or related information from public databases. The typical and major TCM patterns (indicated as A, B, and C in the middle left) can be determined based on an expert consensus or literature analysis. In the right of the diagram, the most commonly used TCM herbal combinations for the treatment of a disease with specific TCM patterns can be found using text mining based on publications from SinoMed database (indicated as A, B, and C, middle right). All of the targeted proteins for the active compounds in the TCM herbal formula can be obtained in PubChem, and these targeted proteins can be used to build up the pharmacological networks for potential multiple-compound drug candidates from the herbal formulas (lower right). By matching the pharmacological networks of herbal compound combinations from the herbal formula with the disease-pattern molecular network, those well-matched compound combinations might be found for new drug candidates (capsule 1, 2, and 3 in lower part).

Based on the integration of the biological network of a disease with specific TCM patterns and the pharmacological network of TCM herbal formulas, TCM-based pharmacology network could lead to a new approach for the multiple compound new drug discoveries.  Figure 1 shows a diagram illustrating the role of the combination of TCM pattern networks in a disease and TCM herbal formula networks in the new drug discovery. The biological network in a disease can be divided into two parts: the biological network of the disease shared by all TCM patterns in the disease, and the TCM pattern network of the disease. The common shared disease biological network can also be divided into subnetworks, and the biological network of the TCM pattern can be also divided into different subnetworks. We can then determine the pharmacological networks of multiple-compound drug candidates (such as candidate drug A and B in the lower part of Figure 1). Drug A, with its pharmacological networks, can regulate a part of the shared disease networks and all three of the TCM pattern (A) networks. Thus, drug A could be developed as an effective treatment for the disease categorized in TCM pattern (A). Similarly, drug B could be developed as an effective treatment for the disease categorized in TCM pattern (B). Therefore, building up the molecular networks of TCM patterns in a specific disease and herbal formula-based pharmacological network could lead to a new strategy of multiple compound drug discoveries. Furthermore, TCM pattern classification could specify the therapeutic scope of a drug candidate. TCM herbal formulas, with multiple compounds and clinically proven effectiveness when used in treating the corresponding TCM pattern in a disease, could be an important source for the new drug discovery. The basic process of TCM network pharmacology-based multiple-component drug discovery includes several steps (Figure 2), mainly the build-up of the disease-TCM pattern molecular networks and the pharmacological networks of the multiple-compound drug candidates from the TCM herbal formulas, and then merging of the multiple-compound drug candidate pharmacological networks with disease-TCM pattern molecular networks. If the pharmacological networks of multiple-compound drug candidates from the TCM formula (indicated as A, B, and C in the right of Figure 2) can be matched to the disease-TCM pattern networks (indicated as A, B, and C in the left of Figure 2), then new drugs (indicated as 1, 2, and 3 with capsules in the bottom of Figure 2) might be discovered.

To give an example of this, take RA for instance. Following the TCM clinical practice, the RA patients can be classified into two main patterns: the cold and hot patterns. The gene expression profiles of blood cell from typical cold and hot pattern RA patients were performed to obtain a systematic view of the molecular signatures separately. The differentially expressed candidate genes from microarray chips were explored using DAVID, GeneSpring, and ingenuity pathway analysis (IPA) software, to analyze the protein-protein interactions (PPI)-related network. Thus, the molecular network of the RA-cold and -hot pattern based on genomics data could be identified. In addition, many public databases are available for disease-related network analysis. In particular, the PubMed, a free service, provides an access to the medline database of citations, abstracts, and some full-text articles on life sciences and biomedical topics. Take all these into consideration, a relatively complete molecular network of the RA-cold and -hot pattern could be achieved.

**Figure 3:**
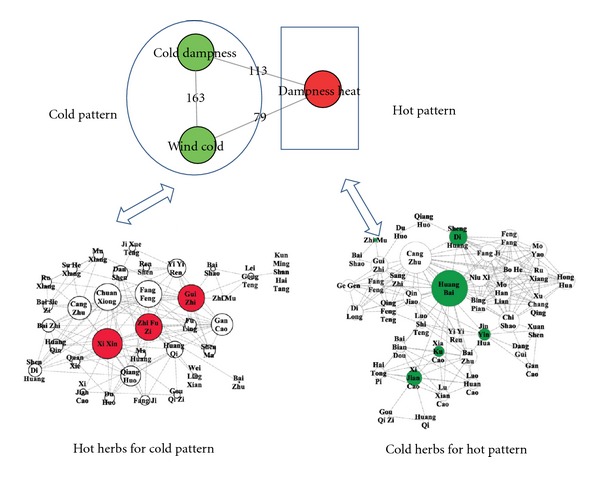
The common herbal formula for the treatment of RA with TCM cold and heat pattern obtained by text mining. All publications about clinical trials in SinoMed were collected, and common combinations of herbs (herbal formula) for the treatment of RA with TCM cold and heat pattern were found.

**Figure 4:**
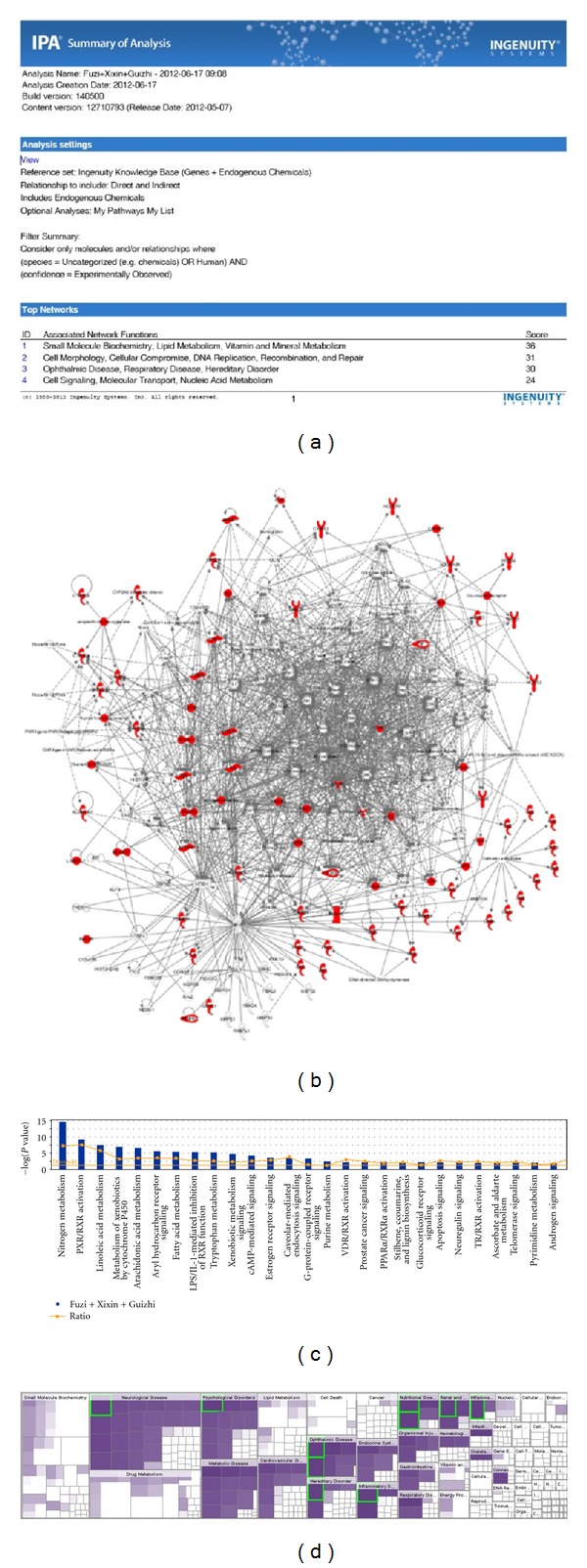
The summary on the network based on the protein targets of Fuzi, Xixin, and Guizhi built up with IPA software. (a) The summary of network analysis results; (b) the merged networks of the protein targets; (c) the canonical pathways related with the protein targets; (d) the hot map of biofunctions related with the protein targets.

For new drug discovery, the major cold or hot patterns in RA are used to build up the molecular networks for merging with the herbal formula pharmacological network. The following questions are that how to find out best potential herbal formula candidates.  Figure 3 showed the potential herbal formula used for cold pattern treatment of RA, and the potential herbal formula used for heat pattern of RA in TCM by text mining. With the support from PubChem bioassay [39], we can study polypharmacological behavior in the PubChem collection via cross-assay analysis [40–42], which can be an important source of drug discovery. Based on PubChem bioassay, researchers can develop a network representation of the assay collection and then apply a bipartite mapping between this network and various biological networks as well as artificial networks (i.e., drug-target network). Mapping to a drug-target network allowed researchers to prioritize new selective compounds, while mapping to other biological networks enabled them to observe interesting target pairs and their associated compounds in the context of biological systems [40]. This approach could be a useful way to build up and investigate the pharmacological network for the multiple compound new drug candidates. As a case, we have chosen protein targets of the compounds in the herbal formula consisting of *Radix Aconiti Praeparata* (Fuzi), *Herba Asari* (Xixin), and *Ramulus Cinnamomi* (Guizhi) which were found good for the treatment of RA with cold pattern by text mining, then pharmacological networks for the multiple compound new drug candidates were built up by IPA (ingenuity pathway analysis software) and protein-protein interaction analysis after collecting their target proteins from PubChem. Furthermore, the functions of the networks and the relationships between the herbal formula networks and disease-pattern networks were analyzed to find new drug candidates for the cold pattern of RA in TCM. As shown in the result of text mining, there were 78 target proteins in the herbal formula (the detail information of every target protein was shown in Table 1). We uploaded the total 78 target proteins to the IPA software online and built up the molecular networks of those target proteins. The analysis results of IPA of the target proteins of the herbal formula (including Fuzi, Xixin, and Guizhi) were shown in Figure 4, in which there were shown the summary of analysis results (Figure 4(a)), the merged network (Figure 4(b)), the canonical pathways (Figure 4(c)), and the hot map of biofunctions related with protein targets of the herbal formula by IPA platform (Figure 4(d)).

Actually, it is obvious that the literature-derived network is relatively crude and redundant for the main reason of the quality control in the text/data mining approaches. Regarding this, it is important to define criteria of literature included and excluded. On the other hand, it is helpful to combine literature mining and *omics* analysis, such as literature mining combined microarray analysis system (LMMA system) [43]. In further, integrating both the experimental data and the literature knowledge seems to be an effective way to reduce noises of data in biological network modeling [44].

## 5. Merging the Molecular Disease Network with the Pharmacological Network of the Candidate Drugs

**Figure 5:**
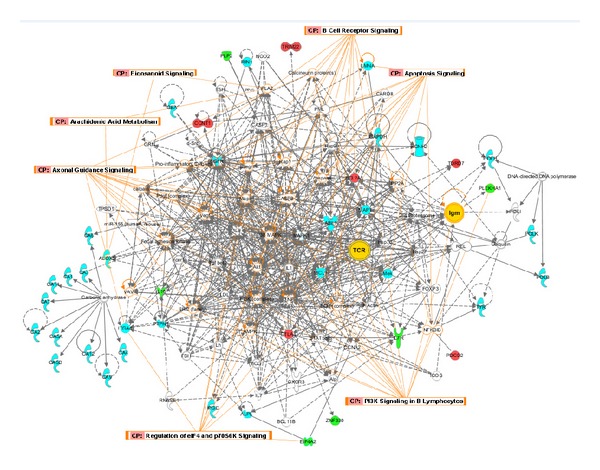
The merged networks of identified differentially expressed genes in RA-cold pattern and protein targets of Fuzi, Xixin, and Guizhi. Red color shows the upregulated genes in RA-cold pattern compared with health; green: the downregulated genes in RA-cold pattern compared with health; Blue color shows the protein targets of Fuzi, Xixin, and Guizhi; yellow color shows the common molecular both in networks of RA-cold pattern and networks of protein targets of Fuzi, Xixin, and Guizhi. (CP: the common canonical pathways related with differentially expressed genes in RA-cold pattern and protein targets of Fuzi, Xixin, and Guizhi both; orange lines: the molecular involved in the common canonical pathways.)

Recently, the essence of life had increasingly been studied from a systems perspective across different scientific disciplines [45]. Plenty of work had been done to provide the practical frameworks for applying “systems thinking” to human diseases and drug discovery [45–47]. The published report showed the relationships between drug targets and disease-gene products, which measured the shortest distance between both sets of proteins in current models of the human protein-protein interaction (PPI) network [4]. Significant differences in distance were found between etiological and palliative drugs, and recent trend toward more rational drug design was observed in the research. Indeed, the method of using the concepts of network biology to integrate data of drug targets and disease-related genes or proteins had been an important way for no matter the discovery of new drug, or repurposing of old drugs. For example, we merged the networks of protein targets of Fuzi, Xixin, and Guizhi and the network of identified differentially expressed genes in RA with TCM cold pattern versus health. As shown in Figure 5, two common molecular (TCR and IgM) and seven common canonical pathways were all found related with the two networks. We considered that the common molecular and canonical pathways might be the potential therapeutic targets of Fuzi, Xixin, and Guizhi to treat RA with cold pattern.

**Figure 6:**
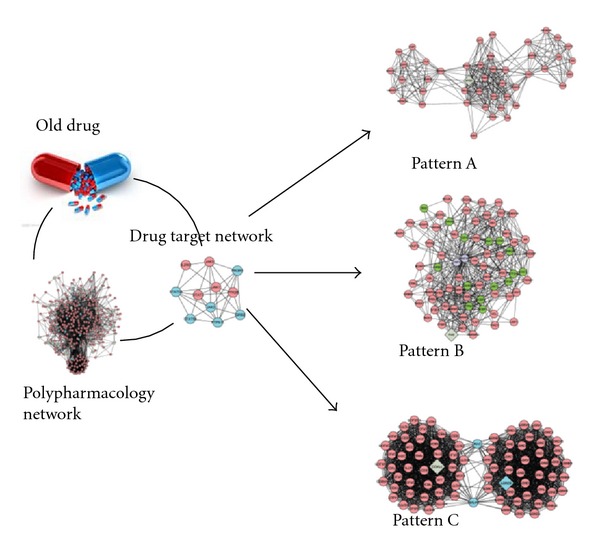
A conceptual paradigm for the repurposing of old drugs based on TCM network pharmacology. The pharmacological networks of an old drug can be built up with bioinformatics approaches (left). By merging the pharmacological networks of the old drug with the disease-TCM pattern molecular network (indicated with (A), (B), and (C) in the right), a better indication based on TCM pattern classification might be found.

On the other hand, drug repurposing, which is the use of established drugs for new indications, would be realized with network pharmacology approaches. Development of a new pharmaceutical product requires at least from 10 to 15 years and costs from $500 million to $2 billion [48–50], yet the number of new drugs approved by the FDA has been declining year by year [51]. Existing drugs already have clinical data and therefore require much less time and money to be approved for a new indication [52]. Researchers have proposed inverse docking models as a novel method to evaluate previously approved drugs for new therapeutic indications [53–55]. Methotrexate (MTX) and sulfasalazine (SSZ) combination therapy is a common treatment for RA, and we found that this combination was more effective for treating RA patients with the TCM cold pattern [56, 57]. In order to find the biological mechanism with network pharmacology, the pharmacological networks of MTX and SSZ were matched with the molecular network of RA with TCM cold pattern, and the network-based pharmacological mechanism result supports the clinical finding [58]. Similarly we can apply the model to screen other existed drug and see which TCM pattern or indication would be better for the drug. Thus we propose a strategy that uses TCM-based network pharmacology for repurposing a marketed “old” drug (Figure 6). Briefly, the pharmacological networks of the marketed drugs can be built based on the information about the pharmacological activity of these drugs from established databases. By matching the pharmacological network of the old drugs and the TCM pattern molecular networks in the disease, we can determine which subgroup of patients would be better candidates for the drugs. In the right of Figure 6, TCM pattern (A), (B), and (C) indicate the molecular networks for the disease with TCM pattern (A), (B), and (C), respectively. If the pharmacological network of the marketed drug can be matched with pattern (A), then the marketed drug could be further investigated clinically for the treatment of the disease with TCM pattern (A). Similarly, we can find new indications for other marketed drugs. Thus, the marketed “old” drug can be regarded as a new drug because it can be used with a new specified indication.

## 6. Perspectives and Conclusions

TCM pattern classification, as a diagnostic approach, could be used to classify patients based on their disease diagnosis in biomedicine. As a result, the TCM pattern could be a potential drug therapeutic target. Additionally, TCM herbal formulas are a vast, promising, and natural resource for drug discovery. More importantly, with their clinically approved effectiveness and safety, they are containing multiple compounds and would be the multiple compound drug candidates. Thus, new drug discovery should put a greater emphasis on TCM pattern classification in certain disease and multiple-compound drug candidates from TCM herbal formulas. We expect that, along the advancement of TCM based network pharmacology, a novel multiple compound drugs would be discovered in the near future.

## Figures and Tables

**Table 1:** Target proteins of Fu Zi, Xi Xin, and Gui Zhi searched in PubChem.

Active compounds	Homosapiens proteins name	GI number
Higenamine	D(2) dopamine receptor	118206

Fuziline	Orexin receptor type 1	222080095

	Caspase 8	2493531
	Vitamin D3 receptor isoform VDRA	63054845
	Microtubule-associated protein tau	92096784
Safrole	Corticotropin-releasing hormone receptor 2	38349113
	Aldehyde dehydrogenase 1 family, member A1	30582681
	Euchromatic histone-lysine N-methyltransferase 2	168985070
	Corticotropin releasing factor-binding protein	30219

	Farnesoid X nuclear receptor	325495553
Methyleugenol	Sentrin-specific protease 8	262118306
	AR protein	124375976

Asaricin	Cytochrome P450 3A4 isoform 1	13435386

	Sentrin-specific protease 8	262118306
	Cytochrome P450 1A2	73915100
Asarinin	Cytochrome P450 2D6 isoform 1	40805836
	Cytochrome P450 2C9 precursor	13699818
	Cytochrome P450 2C19 precursor	4503219

	Transient receptor potential cation channel subfamily A member 1	313104269
	cGMP-specific 3′,5′-cyclic phosphodiesterase	317373261
	Lamin isoform A delta 10	27436948
Cinnamaldehyde	Prothrombin	339641
	Glucocorticoid receptor	311348376
	Aldehyde dehydrogenase 1 family, member A1	30582681
	Chain A, crystal structure of the human 2-oxoglutarate oxygenase Loc390245	221046486
	Glucocerebrosidase	496369

	Thromboxane-A synthase	254763392
	Heat shock protein HSP 90-alpha isoform 2	154146191
	Melanocortin receptor 4	119508433
	Lysosomal alpha-glucosidase preproprotein	119393891
	Alkaline phosphatase, tissue-nonspecific isozyme isoform 1 precursor	116734717
	Tyrosine-protein kinase ABL1 isoform a	62362414
	Nuclear receptor coactivator 3 isoform a	32307126
	Nuclear receptor coactivator 1 isoform 1	22538455
	MPI protein	16878311
	Glyceraldehyde-3-phosphate dehydrogenase isoform 1	7669492
	Glutathione S-transferase omega-1 isoform 1	4758484
	Tyrosinase	401235
	Arachidonate 5-lipoxygenase	126407
	Carbonic anhydrase 2	115456
	Cytochrome P450 2A6	308153612
	Carbonic anhydrase 3	134047703
	Carbonic anhydrase 6	116241278
	Carbonic anhydrase 9	83300925
	Hydroxycarboxylic acid receptor 2	74762622
	Carbonic anhydrase 5B, mitochondrial	8928041
	Carbonic anhydrase 14	8928036
	5-hydroxytryptamine receptor 7	8488960
	Epidermal growth factor receptor	2811086
	Carbonic anhydrase 7	1168744
Cinnamic acid	Carbonic anhydrase 5A, mitochondrial	461680
	Tyrosine-protein phosphatase non-receptor type 1	131467
	Carbonic anhydrase 4	115465
	Carbonic anhydrase 1	115449
	Adenosine receptor A2b	112938
	Lethal(3)malignant brain tumor-like protein 1 isoform I	117938328
	5-hydroxytryptamine receptor 5A	13236497
	potassium voltage-gated channel subfamily H member 2 isoform d	325651834
	DNA polymerase iota	154350220
	DNA polymerase kappa	7705344
	DNA polymerase eta	5729982
	DNA polymerase beta	4505931
	Estrogen receptor beta isoform 1	10835013
	Nuclear receptor subfamily 0 group B member 1	5016090
	Thyroid hormone receptor beta	189491771
	15-hydroxyprostaglandin dehydrogenase [NAD+] isoform 1	31542939
	FAD-linked sulfhydryl oxidase ALR	54112432
	Ras and Rab interactor 1	68989256
	Integrin alpha-4 precursor	67191027
	Chain A, human Ape1 endonuclease with bound abasic DNA And Mn2+ Ion	6980812
	Mcl-1	7582271
	Chain A, structure of human Recq-like helicase in complex with a DNA Substrate	282403581
	Chain A, Jmjd2a tandem tTudor domains in complex with a trimethylated histone H4-K20 peptide	162330054
	Euchromatic histone-lysine N-methyltransferase 2	168985070
	Chain B, the structure of wild-type human Hadh2 bound to Nad+ At 1.2 A	122921311
	Chain A, the structure of wild-type human Hadh2 bound to Nad+ At 1.2 A	122921310
	Bromodomain adjacent to zinc finger domain 2B	6683500
	Carbonic anhydrase 12	5915866
